# A peroxiredoxin, PRDX-2, is required for insulin secretion and insulin/IIS-dependent regulation of stress resistance and longevity

**DOI:** 10.1111/acel.12321

**Published:** 2015-03-23

**Authors:** Monika Oláhová, Elizabeth A Veal

**Affiliations:** Institute for Cell and Molecular Biosciences, Newcastle UniversityFramlington Place, Newcastle upon Tyne, NE2 4HH, UK

**Keywords:** antioxidants, arsenite, FOXO, insulin signalling, Nrf2, oxidative stress

## Abstract

Peroxiredoxins (Prx) are abundant thiol peroxidases with a conserved anti-ageing role. In contrast to most animals, the nematode worm, *Caenorhabditis elegans*, encodes a single cytosolic 2-Cys Prx, PRDX-2, rendering it an excellent model for examining how peroxiredoxins affect animal physiology and ageing. Our previous work revealed that, although PRDX-2 protects against the toxicity of peroxides, enigmatically, *prdx-*2-mutant animals are hyper-resistant to other forms of oxidative stress. Here, we have investigated the basis for this increased resistance. Mammalian FOXO and Nrf2 transcription factors directly promote the expression of a range of detoxification enzymes. We show that the FOXO orthologue, DAF-16, and the Nrf2 orthologue, SKN-1, are required for the increased stress resistance of *prdx-2*-mutant worms. Our data suggest that PRDX-2 is required for normal levels of insulin secretion and hence the inhibition of DAF-16 and SKN-1 by insulin/IGF-1-like signalling (IIS) under nutrient-rich conditions. Intriguingly, loss of PRDX-2 increases DAF-16 and SKN-1 activities sufficiently to increase arsenite resistance without initiating other IIS-inhibited processes. Together, these data suggest that loss of peroxiredoxin function may increase stress resistance by reducing insulin secretion, but that further changes in insulin signalling are required for the reprogramming of development and fat metabolism. In addition, we reveal that the temperature-dependent prolongevity function of PRDX-2 is required for the extended lifespan associated with several pathways, including further reductions in IIS.

## Introduction

There is increasing evidence that reactive oxygen species (ROS) play diverse roles in normal physiology and disease. Reactive oxygen species can cause potentially lethal levels of cell damage. However, over recent years, it has become apparent that, although many diseases and ageing are associated with increased oxidative damage, ROS can have positive roles in slowing the ageing process (Yang & Hekimi, 2010; Zarse *et al*., 2012; Martins *et al*., 2014). Consequently, there remains intense interest in understanding how ROS influence ageing.

As abundant peroxidases, peroxiredoxins are important for ROS responses. Peroxiredoxins also have conserved prolongevity roles (Neumann *et al*., 2003; Olahova *et al*., 2008; Iraqui *et al*., 2009; Lee *et al*., 2009a; Molin *et al*., 2011). The thioredoxin peroxidase activity of the yeast homologue of Prdx1, TSA1, is required for the increased replicative lifespan associated with caloric restriction (Molin *et al*., 2011). Indeed, overexpression of 2-Cys Prx extends the lifespan of yeast and flies (Lee *et al*., 2009a; Molin *et al*., 2011). However, the mechanisms by which Prx slow the ageing process are poorly understood. For instance, yeasts lacking the peroxiredoxin Tsa1 have a shortened replicative lifespan although, surprisingly, telomere length is maintained more effectively than in wild-type cells (Lu *et al*., 2013). In mammals, cytosolic 2-Cys Prx (Prdx1 and Prdx2), orthologous to TSA1 in yeast, have been shown to have nonredundant biological functions, for example in preventing the development of malignant tumours and supporting the production and maintenance of red blood cells in mice (Lee *et al*., 2003; Neumann *et al*., 2003; Rhee & Woo, 2011). However, functional redundancy between Prdx1 and Prdx2 has prevented the detailed examination of how these Prx affect mammalian physiology and the development of age-associated diseases.

*Caenorhabditis elegans* lacking the single cytosolic typical 2-Cys Prx are short-lived and exhibit signs of accelerated ageing (Olahova *et al*., 2008). However, intriguingly, the prolongevity function of PRDX-2 is only apparent at lower temperatures, which specifically increases the lifespan of wild-type animals (Isermann *et al*., 2004; Olahova *et al*., 2008). Notably, although intestinal expression of PRDX-2 provides important protection against the toxicity of peroxides and other oxidants, it does not increase the lifespan (Olahova *et al*., 2008). Indeed, paradoxically, loss of PRDX-2 from other tissues is apparently responsible for both the short lifespan of *prdx-2-*mutant animals and their increased resistance to some oxidants, for example arsenite (Olahova *et al*., 2008). Hence, the relationships between the tissue-specific functions of PRDX-2 in stress resistance and PRDX-2’s temperature-dependent, anti-ageing function remain unclear.

Glutathione and phase 2 detoxification reactions are important for the detoxification of arsenite (Liao & Yu, 2005). Our previous work revealed that *prdx-2*-mutant animals express elevated levels of phase 2 detoxification genes, including *gcs-1* that encodes a key enzyme in glutathione biosynthesis. The stress-induced expression of *gcs-1* requires the activation of the p38-related MAPK PMK-1, which phosphorylates and increases the activity of SKN-1 (Inoue *et al*., 2005). However, the arsenite-induced activation of the PMK-1 MAPK is impaired in *prdx-2-*mutant animals and the increased resistance of PRDX-2-deficient animals to arsenite is only partly dependent on SKN-1 (Olahova *et al*., 2008). Together, these data suggested that loss of PRDX-2 leads to the activation of an unidentified mechanism(s) that is able to bypass the canonical SEK-1-/PMK-1-/SKN-1-signalling pathway to increase arsenite tolerance (Olahova *et al*., 2008).

**Fig 1:**
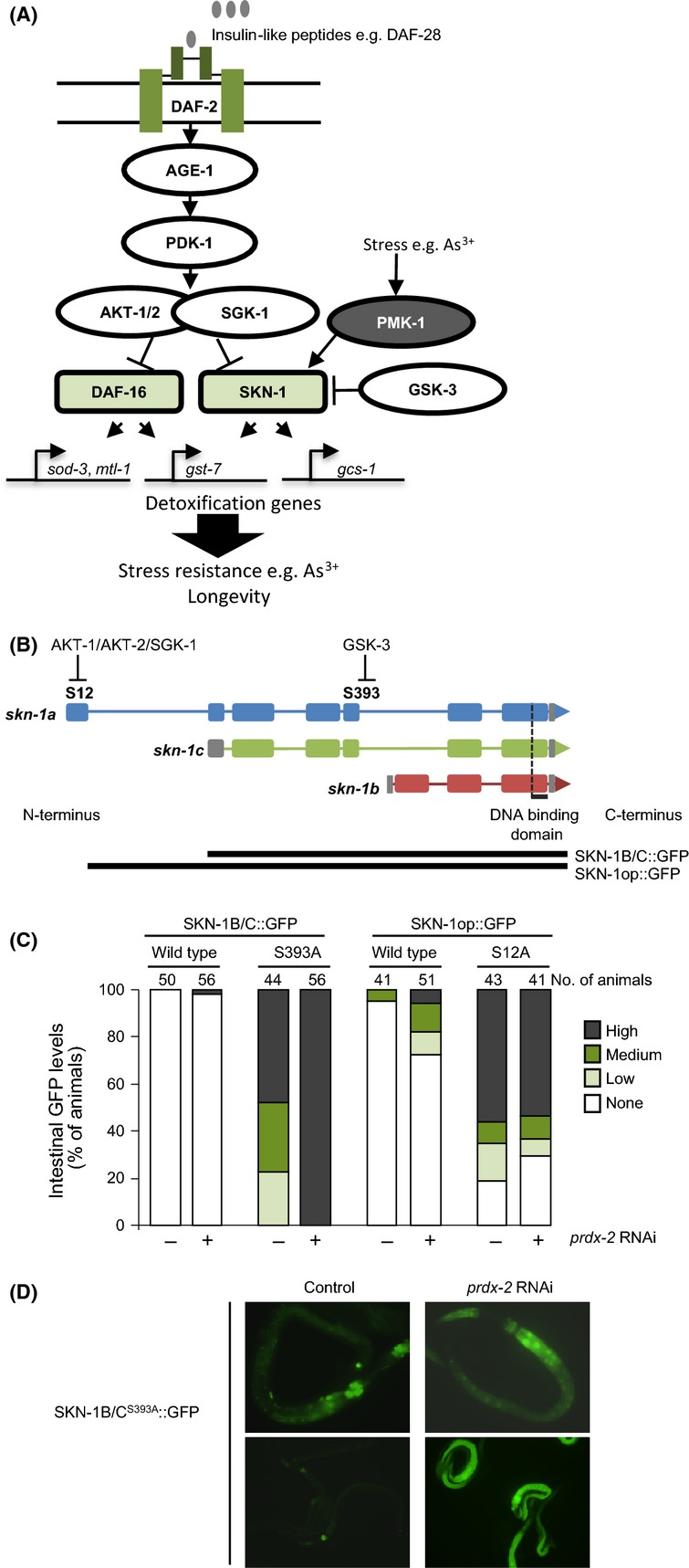
PRDX-2 is required for insulin-dependent inhibition of SKN-1. (A) DAF-16 and SKN-1 transcription factors activate distinct and overlapping detoxification/antioxidant genes to increase stress resistance. Insulin/IGF-1-like signalling (IIS) through the DAF-2 insulin receptor activates AKT-1/AKT-2/SGK-1 kinases, which phosphorylate DAF-16 and SKN-1. The activity of AKT/SGK kinases is stimulated by insulin through the activation of the PI3 kinase AGE-1 and PDK-1. AKT-1/2-mediated phosphorylation of DAF-16 and AKT-1/2/SGK-1-mediated phosphorylation of SKN-1 inhibit the nuclear accumulation of these transcription factors. When insulin signalling is reduced, and following phosphorylation by stress-activated MAPK, for example PMK-1, this inhibition is overcome. In response to stress, for example arsenite, the PMK-1 MAPK is activated and phosphorylates SKN-1 and DAF-16, increasing their activity (Inoue *et al*., 2005; Kondo *et al*., 2005). This allows the nuclear accumulation of DAF-16 and SKN-1, which together orchestrate the expression of a battery of oxidative stress defence genes (Murphy *et al*., 2003; Tullet *et al*., 2008; Oliveira *et al*., 2009) (for a review see Lapierre & Hansen, 2012). SKN-1 is also inhibited by GSK-3-mediated phosphorylation (An *et al*., 2005). (B) Graphical depiction of the *skn-1* gene structure to show the different SKN-1 isoforms and serines 12, in SKN-1A, and 393, in SKN-1A and C, that are subject to inhibitory phosphorylation by AKT-1/2/SGK-1 (Tullet *et al*., 2008) and GSK-3 (An *et al*., 2005), respectively. Exons are represented by the broad boxes. (C) Loss of PRDX-2 increases the levels of SKN-1::GFP or SKN-1^S393^^A^::GFP in intestinal nuclei but not the levels of a partially active SKN-1A mutant in which the AKT phosphorylation site (S12) is substituted with alanine (Tullet *et al*., 2008). Intestinal SKN-1::GFP levels were scored as ‘none’, ‘low’, ‘medium’ or ‘high’ in L2/L3 larval stage control (pL4440) or *prdx-2* RNAi (pL4440 + *prdx-2*)-treated animals expressing the indicated SKN-1::GFP extrachromosomal arrays. Chi-square test, SKN-1B/C::GFP control compared with *prdx-2* RNAi *P* = 0.34, SKN-1B/C^S^^393A^::GFP control compared with *prdx-2* RNAi *P* < 0.0001, SKN-1op::GFP control compared with *prdx-2* RNAi *P* = 0.028 and SKN-1op^S12A^::GFP control compared with *prdx-2* RNAi *P* = 0.49. The experiment was repeated at least three times with similar results. A representative experiment is shown. (D) Upper panels show SKN-1^S393A^::GFP in representative control (left panel) and *prdx-2* RNAi-treated (right panel) animals. Lower panels show images of several control (left panel) or *prdx-2* RNAi-treated (right panel) animals taken at identical exposure.

Here, we have investigated the basis for the increased arsenite resistance and shortened lifespan of *prdx-2-*mutant *C. elegans* at 15 °C. Interestingly, our data suggest that loss of PRDX-2 increases arsenite resistance by reducing the levels of secreted insulin and hence increasing the intestinal activity of both SKN-1 and the FOXO transcription factor DAF-16 (Fig.1A). Intriguingly, our demonstration that further reductions in insulin signalling are required to produce changes in metabolism, development or longevity implies differential regulation of specific physiological responses. Moreover, our discovery that PRDX-2 is required for insulin secretion reveals a new physiological role for a peroxiredoxin, as well as provides an explanation for the unexpected role of this peroxidase in limiting *C. elegans* stress resistance.

## Results

### PRDX-2 is required for insulin/IIS-dependent inhibition of SKN-1

Loss of *prdx-2* increases the expression of SKN-1-regulated phase 2 detoxification genes, including *gcs-1* that is important for resistance to arsenite (An & Blackwell, 2003; Liao & Yu, 2005; Olahova *et al*., 2008). Hence, to test whether the increased expression of these phase 2 genes in *prdx-2*-mutant animals might reflect increased SKN-1 activity, we examined the nuclear levels of SKN-1::GFP in animals treated with *prdx-2* RNAi. The effect of *prdx-2* RNAi was examined on transgenic lines containing arrays expressing different wild-type SKN-1::GFP isoforms. SKN-1B/C::GFP encodes both the SKN-1B and SKN-1C isoforms, whereas SKN-1op::GFP also encodes SKN-1A (Fig.1B) (Tullet *et al*., 2008). Nuclear levels of SKN-1::GFP are barely detectable in the intestinal nuclei of animals maintained under normal conditions. However, *prdx-2* RNAi caused SKN-1::GFP to be detected in intestinal nuclei of a small number of SKN-1B/C::GFP animals and significantly increased the number of SKN-1op::GFP animals containing nuclear SKN-1::GFP (Fig.1C). This suggests that loss of PRDX-2 increases the activity of SKN-1, particularly the SKN-1A form (Tullet *et al*., 2008).

SKN-1 is positively regulated by PMK-1-dependent phosphorylation (Inoue *et al*., 2005) (Fig.1A). Previously, we had shown that the arsenite-induced activation of PMK-1 was impaired in the *prdx-2* mutant, ruling out increased PMK-1-mediated activation of SKN-1 as responsible for the increased SKN-1 activity in *prdx-2-*mutant animals (Olahova *et al*., 2008). However, SKN-1 is also negatively regulated by a number of mechanisms, including GSK-3- and AKT-1-/AKT-2-/SGK-1-mediated phosphorylation of specific serine residues (An *et al*., 2005; Tullet *et al*., 2008) (Fig.1A,B). To determine whether the increased levels of SKN-1 in intestinal nuclei might be due to reduced insulin-dependent phosphorylation by AKT-1/SGK-1, or alternatively reduced phosphorylation by GSK-3, we compared the nuclear levels of mutant forms of SKN-1, SKN-1B/C^S393A^::GFP and SKN-1op^S12A^::GFP in which serines required for GSK-3- and insulin-mediated inhibition, respectively, are substituted with alanine (Fig.1A,B) (An *et al*., 2005; Tullet *et al*., 2008). Consistent with previous work, SKN-1B/C^S393A^::GFP, that retains AKT/SGK sites but lacks the GSK-3 phosphorylation site, was detected in some (‘low’ and ‘medium’) or all (‘high’) intestinal nuclei in control animals (Fig.1C,D) (An *et al*., 2005). However, SKN-1B/C^S393A^::GFP was present at high levels in every intestinal nucleus in *prdx-2* RNAi-treated animals (Fig.1C,D). This suggests that the inhibition of SKN-1 by PRDX-2 does not require GSK-3-mediated phosphorylation of SKN-1. In contrast, loss of PRDX-2 did not increase the nuclear levels of SKN-1op^S12A^::GFP in which a key IIS-inhibited phosphorylation site is substituted with alanine (Fig.1B,C) (Tullet *et al*., 2008). This raised the intriguing possibility that PRDX-2 might reduce SKN-1 activity by promoting the IIS-dependent inhibition of SKN-1 (Fig.1A).

### PRDX-2 is required for insulin secretion

**Fig 2:**
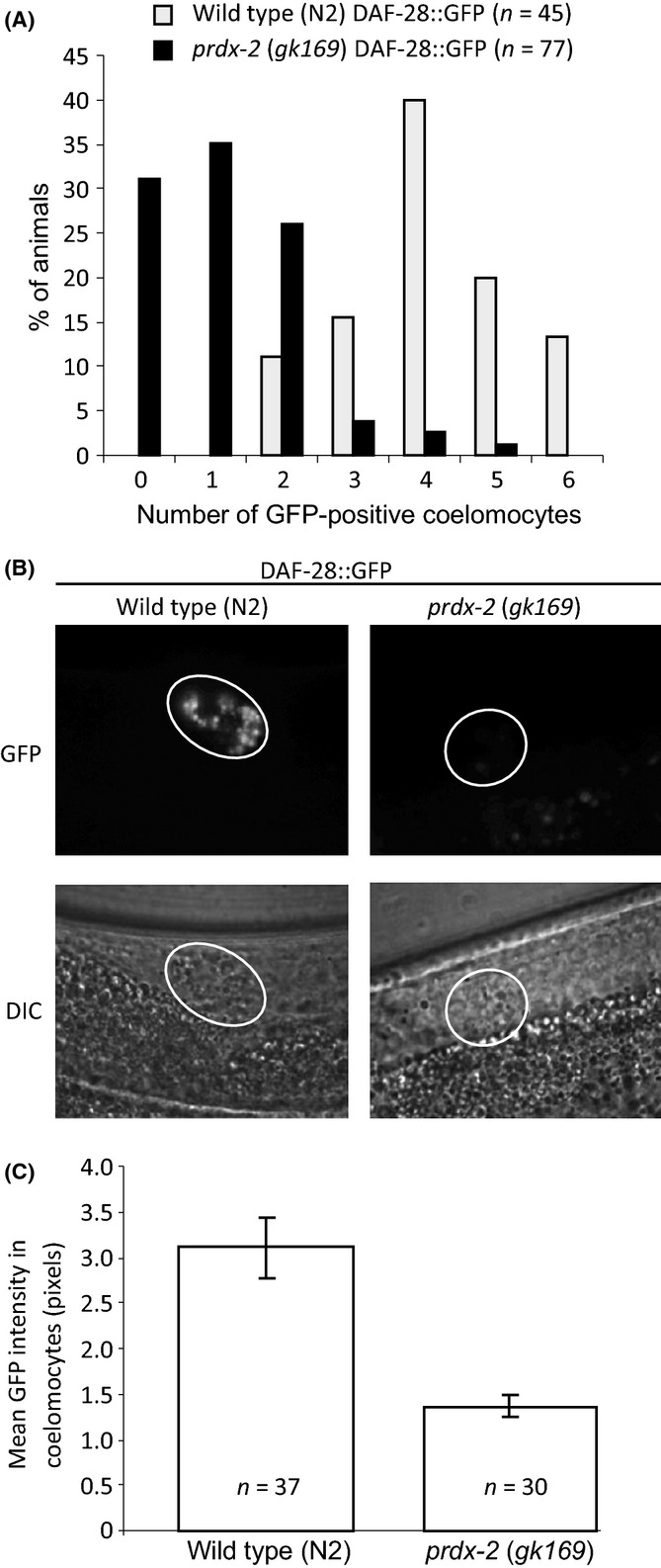
PRDX-2 is required for insulin (DAF-28::GFP) secretion under well-fed conditions. (A) There is a significant decrease in the number of coelomocytes containing visible levels of DAF-28::GFP in *prdx-2* (*gk169*)-mutant animals compared with wild-type (N2) animals (chi-square test *P* = 4.8 × 10^−16^). (B) Representative fluorescent (GFP) images, with identical exposures, reveal lower levels of DAF-28::GFP in GFP-positive coelomocytes from wild-type N2 and *prdx-2* (*gk169*) 1-day-old adults expressing DAF-28::GFP. Differential interference contrast light (DIC) images are also shown. Coelomocytes are indicated by white circles. Gut autofluorescence visible outside of circle is, as expected (Olahova *et al*., 2008), increased in *prdx-2-*mutant animals. (C) Relative pixel intensities of coelomocytes GFP contents indicate substantially reduced DAF-28::GFP levels in *prdx-2* (*gk169*)-mutant coelomocytes compared with wild-type N2 animals. DAF-28::GFP fluorescence in coelomocytes was quantified by measuring the mean pixel density in fluorescent images of wild-type N2 and *prdx-2* (*gk169*)-mutant animals with identical exposures. Error bars represent the standard deviation from the mean. *n* refers to the number of animals in each group. Student’s *t*-test compares wild-type with *prdx-2* mutant *P* < 0.0001. The experiment was repeated with similar results.

To determine whether the increased nuclear SKN-1 levels in PRDX-2-deficient animals reflected reduced insulin signalling, we began by testing whether loss of PRDX-2 affected the release of insulin ligands. Under favourable conditions, the insulin-like neuropeptide DAF-28 is highly expressed and acts as a DAF-2 receptor agonist, increasing IIS activity. To determine the ability of *prdx-2* mutants to secrete insulin under well-fed conditions, we employed a strain expressing DAF-28::GFP. In well-fed animals, DAF-28::GFP is secreted from the ASJ and ASI neurons into the pseudocoelom from which it acts as a ligand for the insulin receptor, DAF-2, promoting IIS in many tissues (Fig.1A) (Kao *et al*., 2007). *Caenorhabditis elegans* contains six macrophage-like scavenging cells (coelomocytes) that continuously take up the pseudocoelomic fluid (Fares & Greenwald, 2001a). Hence, the GFP fluorescence intensity in coelomocytes has been established as a reliable *in vivo* measure of the secretion of GFP-tagged neuropeptides, including DAF-28::GFP, in intact animals (Fares & Greenwald, 2001a). Loss of PRDX-2 does not have the strong larval arrest phenotype associated with mutants in which insulin secretion is severely impaired (Kao *et al*., 2007). Nevertheless, 30% of *prdx-2*-mutant animals expressing DAF-28::GFP lacked any GFP-positive coelomocytes and only 35% of *prdx-2-*mutant animals contained more than one coelomocyte with detectable GFP flu-orescence (Fig.2A). In contrast, at least two GFP-positive coelomocytes were easily detected in every wild-type DAF-28::GFP animal (Fig.2A). Moreover, quantification revealed a significant decrease in the intensity of DAF-28::GFP fluorescence in GFP-positive coelomycetes in *prdx-2-*mutant animals compared with wild-type animals (Fig.2B,C). Importantly, loss of PRDX-2 did not reduce the levels of a muscle-expressed, secretion-targeted GFP (ssGFP) that accumulated in coelomocytes (Fig. S1A, Supporting information). This indicates that the lower levels of DAF-28::GFP present in PRDX-2-deficient coelomocytes (Figs2 and S1B) do not reflect a general impairment in protein secretion or defective coelomocyte endocytosis. It was possible that the reduced insulin secretion reflected lower levels of insulin synthesis in *prdx-2-*mutant worms. However, the intensity of DAF-28::GFP in the neurons of control and PRDX-2-deficient animals was similar (Fig. S2A,B). Moreover, there was no reduction in the total levels of DAF-28::GFP protein detected by immunoblotting (Fig. S2C). Together, these data strongly suggest that PRDX-2 is required for the normal neuronal secretion of DAF-28::GFP into the pseudocoelom.

### Loss of PRDX-2 increases arsenite resistance by increasing both SKN-1 and DAF-16 activities

**Fig 3:**
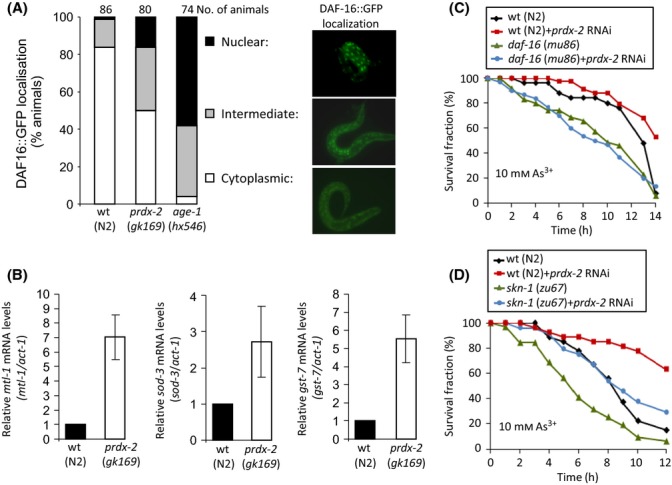
Loss of PRDX-2 increases arsenite resistance by increasing both SKN-1 and DAF-16 activities. (A) Loss of *prdx-2* causes nuclear accumulation of DAF-16. The localization of a DAF-16::GFP fusion protein was assessed in L2/L3 larval stage wild-type, *prdx-2* (*gk169*)- and *age-1* (*hx584*)-mutant animals expressing daf-16a-16::GFP. PRDX-2 deficiency caused nuclear accumulation of daf-16a::GFP in the intestinal nuclei (chi-square test *P* = 7.0 × 10^−5^). *n* refers to the number of worms examined in each group in the representative experiment shown. The experiment was repeated several times at 15 °C with similar results. (B) *prdx-2-*mutant animals contain increased levels of mRNA for *mtl-1*, *sod-3* and *gst-7* compared with wild-type (N2) animals. mRNA levels were calculated relative to control (*act-1*) mRNA in at least six independently prepared RNA samples. Each panel depicts the levels of a particular mRNA in *prdx-2* mutant normalized to wild-type (N2). Error bars represent the SEM. For individual experiments and statistical analysis, see Table S1. (C, D) The survival of L4 larval stage wild-type (N2), *daf-16 (mu86)-* and *skn-1 (zu67)-*mutant animals microinjected with *prdx-2* dsRNA was monitored on NGM-L plates containing 10 mm sodium arsenite at indicated time points. (C) Loss of *prdx-2* significantly increased the arsenite resistance of wild-type but not *daf-16* (*mu86*)-mutant animals. Log-rank analysis on wild-type (N2) control compared with *daf-16* (*mu86*) *P* = 0.090, wild-type (N2) control compared with wild-type (N2) + *prdx-2* RNAi *P* = 0.002 and wild-type (N2) + *prdx-2* RNAi compared with *daf-16* (*mu86*) *+ prdx-2* RNAi *P* < 0.0001. (D) *prdx-2* RNAi produces a greater increase in the arsenite resistance of wild-type than *skn-1* (*zu67*)-mutant animals. Log-rank analysis on wild-type (N2) vs. wild-type (N2) *+ prdx-2* RNAi *P* < 0.0001, *skn-1* (*zu67*) vs. *skn-1* (*zu67*)* *+ *prdx-2* RNAi *P* = 0.004. The survival assays in (C) and (D) were repeated at least three times with similar results.

Like SKN-1, the FOXO transcription factor DAF-16 is also inhibited by IIS-dependent phosphorylation (Fig.1A). In wild-type animals, DAF-16 is normally maintained in the cytoplasm by inhibitory phosphorylation from the IIS-activated AKT kinases that are activated downstream of AGE-1 and PDK-1 (Fig.1A). Hence, to test whether loss of PRDX-2 also increased DAF-16 activity, we examined the localization of GFP in transgenic wild-type and *prdx-2-*mutant animals expressing DAF-16::GFP (Lin *et al*., 2001). Although smaller than the increase observed in *age-1-*mutant animals, there was a significant increase in the nuclear localization of DAF-16::GFP in *prdx-2*-mutant animals (Fig.3A). Consistent with increased DAF-16 activity, qRT–PCR analysis confirmed that the endogenous expression of several DAF-16-activated genes (*mtl-1*, *sod-3*, *gst-7*) (Murphy *et al*., 2003) was increased in *prdx-2*-mutant animals (Fig.3B and Table S1, Supporting information). A *sod-3p*::*gfp* reporter was also upregulated in *prdx-2* RNAi-treated animals in a manner that was partly dependent on DAF-16 (Fig. S3). Moreover, *prdx-2* RNAi failed to increase the arsenite resistance of *daf-16-*mutant animals, suggesting that DAF-16 is required for the increased arsenite resistance associated with loss of *prdx-2* (Fig.3C). However, reduced IIS and loss of PRDX-2 also increase the SKN-1 activity (Tullet *et al*., 2008) (Fig.1), which also activates *gst-7* (Oliveira *et al*., 2009) and other genes, such as *gcs-1*, that are important for resistance to arsenite (Liao & Yu, 2005; Oliveira *et al*., 2009). Indeed, the increased arsenite resistance associated with the loss of PRDX-2 is also partially dependent on SKN-1 (Fig.3D) (Olahova *et al*., 2008). Together, these data suggest that elevated DAF-16 and SKN-1 activities mediate the increased arsenite resistance of *prdx-2-*mutant animals.

### Reduced IIS-dependent inhibition of DAF-16 and SKN-1 is sufficient to explain the increased arsenite resistance associated with loss of PRDX-2

**Fig 4:**
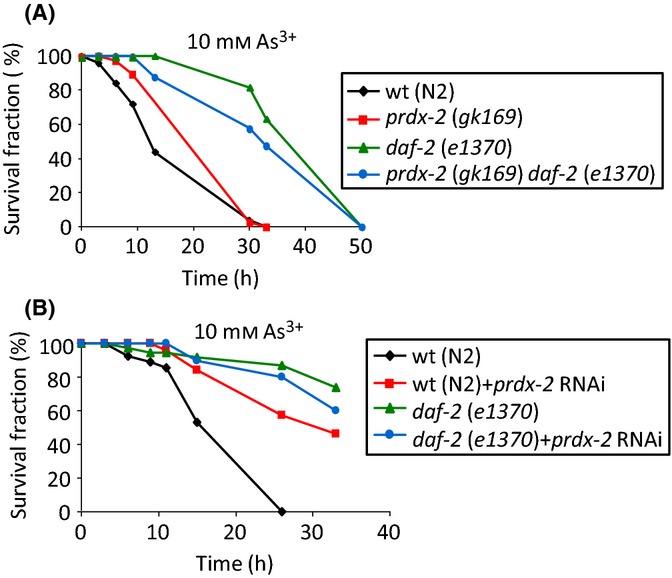
The increased arsenite resistance associated with loss of PRDX-2 requires normal levels/activity of the DAF-2 insulin receptor. Loss of DAF-2 further increases the arsenite hyper-resistance of *prdx-2* mutants. The survival rate of L4 larval stage (A) wild-type (N2), *prdx-2* (*gk169*), *daf-2* (*e1370*) and *prdx-2* (*gk169*) *daf-2* (*e1370*) double mutant or (B) control or *prdx-2* RNAi-treated wild-type (N2) or *daf-2* (*e1370*) hermaphrodites on plates containing 10 mm sodium arsenite was monitored at indicated time points. The experiments were repeated three times with similar results. In (A), log-rank analysis on wild-type (N2) compared with *prdx-2* (*gk169*) *P* < 0.001 and *daf-2* (*e1370*) *P* < 0.0001, *prdx-2* (*gk169*) and *daf-2* (*e1370*) *P* < 0.0001. In (B), log-rank analysis comparing control and *prdx-2* RNAi-treated wild-type (N2) *P* < 0.001, comparing *prdx-2* RNAi-treated wild-type (N2) with *prdx-2* RNAi-treated *daf-2* (*e1370*) *P* = 0.276.

To determine whether reduced insulin signalling was sufficient to fully explain the increased arsenite resistance associated with loss of PRDX-2, we examined whether loss of *prdx-2* affected the arsenite resistance of *daf-2* (*e1370*)-mutant animals. The *daf-2* (*e1370*) allele encodes a less active form of the insulin receptor (DAF-2), the function of which is further reduced at 25 °C (Gems *et al*., 1998). As expected, consistent with reduced DAF-2 activity and increased activity of DAF-16 and SKN-1, *daf-2* (*e1370*) animals were hyper-resistant to arsenite-induced stress (Fig.4). Moreover, loss of PRDX-2, by RNAi or loss of gene function (*gk169*), did not cause a further increase in the expression of a *sod-3p*::*gfp* transgene (Fig. S3D) or the arsenite resistance of *daf-2* (*e1370*)-mutant animals (Fig.4). Thus, the increased *sod-3p*::*gfp* expression and arsenite resistance associated with loss of PRDX-2 (Figs3C,D, 4 and S3) (Olahova *et al*., 2008) are dependent on normal levels/activity of the insulin receptor. This is consistent with the underlying defect in DAF-28::GFP secretion, and derepression of DAF-16 and SKN-1, being responsible for the increased arsenite resistance of *prdx-2-*mutant animals. Notably, *daf-2-*mutant animals were more resistant to arsenite than *prdx-2-*mutant animals, suggesting that further derepression of DAF-16 and SKN-1 in these animals might cause a more substantial increase in stress resistance. However, the small, but insignificant, decrease in the arsenite resistance of *daf-2* (*e1370*) animals lacking PRDX-2 raises the possibility that the arsenite-protective function of intestinal PRDX-2 (Olahova *et al*., 2008) might also contribute to the increased arsenite resistance of *daf-2* (*e1370*).

### Further reductions in IIS are required for dauer formation and increased fat storage in *prdx-2-*mutant animals

In addition to the increased stress resistance, lower levels of IIS have profound effects on *C. elegans* development and metabolism. For example, another DAF-16-mediated effect of reduced IIS is increased fat storage (Ogg *et al*., 1997; Perez & Van Gilst, 2008). Notably, the reduction in IIS associated with loss of *prdx-2* was insufficient to increase fat accumulation. Importantly, although *prdx-2* (*gk169*)-mutant animals contained normal levels of fat, the high fat levels in *daf-2* (*e1370*) *prdx-2* (*gk169*) double mutants were similar to those observed in *daf-2* (*e1370*) at 15 °C (Fig. S4), indicating that PRDX-2 is not required for the increased fat synthesis associated with loss of IIS. This suggested that further changes in IIS might be required to increase fat storage.

Insulin signalling also plays an important part in inhibiting dauer formation. The dauer state is a stress-resistant alternative larval form that is induced by dauer pheromone under stressful conditions, such as when animals are overcrowded. When animals containing the *daf-2* (*e1370*) temperature-sensitive allele are maintained at the nonpermissive temperature (25 °C), the increased DAF-16 activity causes induction of dauer even when food is plentiful (Table1) (Henderson & Johnson, 2001; Lee *et al*., 2001; Lin *et al*., 2001). Importantly, *prdx-2*-mutant animals bearing the *daf-2* (e*1370*) allele still entered dauer at 25 °C (Table1), indicating that PRDX-2 is not required for dauer formation. However, despite the increased activity of DAF-16 in *prdx-2-*mutant animals, no dauer animals were observed under normal growth conditions at 15, 20 or 25 °C (data not shown and Table1). At 27 °C, a small proportion of wild-type animals undergo transient dauer formation (< 1%), even when food is plentiful (Ailion & Thomas, 2000). *prdx-2*-mutant animals develop more slowly than wild-type at temperatures above 15 °C (Isermann *et al*., 2004 and our unpublished observations). This, coupled with the asynchrony of development and transient nature of dauer formation at 27 °C (Ailion & Thomas, 2000), made it difficult to quantitatively compare dauer formation in wild-type and *prdx-2*-mutant animals at 27 °C in a reliable way. However, consistent with increased DAF-16 activity in *prdx-2*-mutant animals augmenting the induction of dauer at higher temperatures (Ailion & Thomas, 2000), there appeared to be a small increase in the proportion of *prdx-2*-mutant animals that formed dauer at 27 °C. Nevertheless, in contrast to mutants in IIS pathway components, less penetrant than *daf-2* (*e1370*) but constitutively dauer at 27 °C e.g. *akt-1* (*mg306*) (Ailion & Thomas, 2003; Hu *et al*., 2006), the vast majority (309 of 318) of *prdx-2-*mutant animals still proceeded to adulthood at 27 °C.

**Table 1:** PRDX-2 is not required for the constitutive dauer phenotype associated with reduced DAF-2 activity and slightly suppresses dauer formation in *daf-2* (*e1370*) mutants at 20 °C and *akt-1* (*mg306*) mutants at 25 °C. Wild-type (N2), *prdx-2* (*gk169*)-*, daf-2* (*e1370*)-, *prdx-2* (*gk169*)- and *daf-2* (*e1370*)-mutant adult animals were allowed to lay eggs for 3 h at 15 °C and then removed from plates. The eggs were incubated at 25 or 20 °C, and 48 h later, the number of dauer animals was scored. Data sets are representative of three independent experiments. The increase in % dauer formation in *prdx-2* (*gk169*) *daf-2* (*e1370*) compared with *daf-2* (*e1370*) was not statistically significant (chi-square test *P* = 0.155). The increase in % dauer formation in *prdx-2* RNAi-treated compared with vector control-treated *akt-1* (*mg306*)*-*mutant animals was statistically significant (chi-square test *P* = 0.0001)

Strain	25 °C			20 °C		
	Adults	Dauers	% Dauer	Adults	Dauers	% Dauer
Wild-type (N2)	110	0	0	402	0	0
*prdx-2* (*gk169*)	167	0	0	541	0	0
*daf-2* (*e1370*)	0	137	100	230	6	2.54
*prdx-2* (*gk169*) *daf-2* (*e1370*)	0	137	100	545	27	4.72
Wild-type (N2) vector	99	0	0	–	–	–
Wild-type (N2) *prdx-2* RNAi	101	0	0	–	–	–
*akt-1* (*mg306*) vector	311	2	0.64	–	–	–
*akt-1* (*mg306*) *prdx-2* RNAi	120	9	7.5	–	–	–

However, when we examined the effect of loss of *prdx-2* on mutants in which IIS is reduced, we did observe a slight increase in the number of dauers formed by *daf-2* (*e1370*) mutants at 20 °C (Table1). Furthermore, although loss of *akt-1* function does not reduce IIS sufficiently to constitutively induce dauer formation until temperatures are raised to 27 °C (Ailion & Thomas, 2003; Hu *et al*., 2006), a significantly increased proportion of *prdx-2* RNAi-treated *akt-1-*mutant animals underwent dauer formation at 25 °C (Table1). Taken together, these data suggest that loss of PRDX-2 reduces IIS and increases DAF-16 and SKN-1 activities sufficiently to increase arsenite resistance, but that further reductions in IIS are required to increase fat storage or induce dauer formation.

### PRDX-2 is required for the lifespan-extending effects of reduced insulin signalling

**Fig 5:**
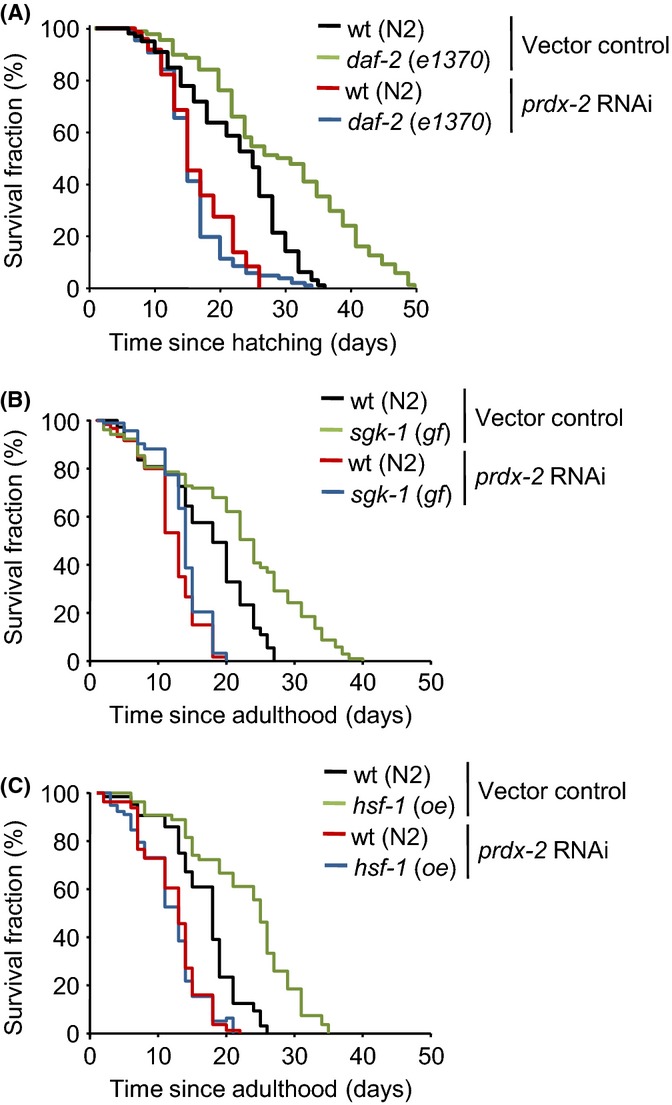
PRDX-2 is required for the life-extending effects of reduced insulin/IGF-1-like signalling. (A) The lifespan of wild-type (N2) and *daf-2 (e1370)* mutants treated with *prdx-2* RNAi or vector control indicates that PRDX-2-depleted *daf-2* (*e1370*) animals were shorter-lived compared with wild-type (N2) and *daf-2* (*e1370*) worms maintained on control plates. (B) The lifespan of wild-type (N2) and *sgk-1gf* (*ft15*) mutants treated with *prdx-2* RNAi or empty vector control RNAi is shown. The extended lifespan of *sgk-1gf* (*ft15*) animals compared with wild-type (N2) on vector control was completely prevented by *prdx-2* RNAi. (C) The lifespans of wild-type (N2) and *hsf-1* (*oe*) animals treated with *prdx-2* RNAi or empty vector control RNAi are shown. The extended lifespan of *hsf-1* (*oe*) animals compared with wild-type (N2) on vector control was ablated by *prdx-2* RNAi. All lifespans were repeated with similar results. Representative experiments are shown. For statistical analysis, see Table S2.

Following seminal discoveries in *C. elegans* (Kenyon *et al*., 1993), it is now well established that reduced insulin signalling is able to delay age-associated declines in tissue function and produce considerable lifespan extensions in animals (Clancy *et al*., 2001; Bluher *et al*., 2003; Holzenberger *et al*., 2003). The increased stress resistance of *C. elegans* with reduced insulin signalling is associated with substantial increases in lifespan that are dependent on DAF-16 and SKN-1 (Lin *et al*., 2001; Tullet *et al*., 2008). In contrast, despite the increased activity of DAF-16 and SKN-1 and resistance to arsenite, at 15 °C, loss of PRDX-2 causes a dramatic shortening of lifespan as a result of accelerated ageing (Olahova *et al*., 2008) (Fig.5, Table S2). Indeed, although *daf-2* (*e1370*)-mutant animals were long-lived at 15 °C, this increase in lifespan was largely ablated by *prdx-2* RNAi (Fig.5A). Importantly, *prdx-2* RNAi did not reduce the lifespan of long-lived *C. elegans* that harbour a mutation in cytochrome *c* reductase *cyc-1*, an important component of the electron transport chain. This indicates that loss of PRDX-2 does not lead to a general sickness or limit lifespan at 15 °C (Fig. S5). Hence, these data suggest that, although loss of PRDX-2 increases DAF-16 and SKN-1 activities (Figs1C,D and 3A,B) and arsenite resistance (Figs3C,D and 4) at 15 °C, PRDX-2 is required for the full extension in lifespan associated with reduced insulin signalling at this temperature.

It was possible that PRDX-2 was important for the increased lifespan-promoting activity of nuclear DAF-16 at 15 °C or, alternatively, acted independently to increase the lifespan. To distinguish between these possibilities, we examined whether PRDX-2 was required for the lifespan extension associated with a gain of function in the SGK-1 kinase that increases the lifespan in a DAF-16-dependent manner (Chen *et al*., 2013). Our discovery that PRDX-2 was essential for the increased longevity associated with the *sgk-1 gf* allele suggested that PRDX-2 acts downstream or independently from SGK-1 to increase lifespan (Fig.5B).

The transcription factor, heat-shock factor, HSF-1, coregulates many DAF-16 targets and is also important for the increased lifespan associated with reduced IIS (Hsu *et al*., 2003; Morley & Morimoto, 2004; Seo *et al*., 2013). Hence, it was possible that the failure of decreased IIS or *sgk-1* gain of function to increase the lifespan of PRDX-2-deficient animals might reflect lower levels of HSF-1 activity in these animals. However, although transgenic overexpression of *hsf-1* increased the lifespan at 15 °C, it failed to increase the lifespan of PRDX-2-deficient animals (Fig.5C). Although the basis of PRDX-2’s essential prolongevity function is unknown, together, these data suggest that PRDX-2 acts independently of IIS to promote increased longevity at 15 °C.

**Fig 6:**
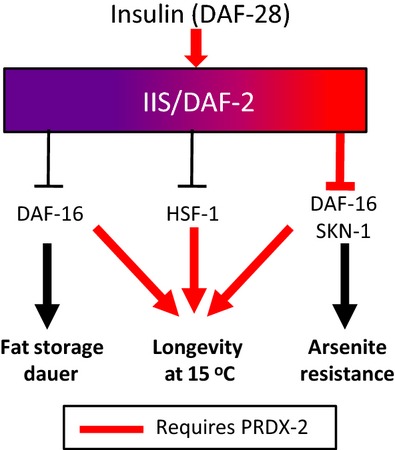
Model for the role of PRDX-2 in regulation of IIS and arsenite resistance. There is considerable complexity in the inputs and outputs of the DAF-2/insulin-like signalling pathway. This complexity is indicated by the coloured shading, with red signifying the aspects which require PRDX-2. PRDX-2 is required for the secretion of at least one insulin-like peptide, the DAF-2 agonist, DAF-28. DAF-2 signalling inhibits the activity of DAF-16, SKN-1 (Fig.1A) and HSF-1. In the absence of PRDX-2, lower levels of insulin (DAF-28) secretion reduce the activity of DAF-2 (IIS) and hence increase the intestinal SKN-1 and DAF-16 activities sufficiently to increase arsenite resistance. However, further changes in IIS are required for DAF-16-dependent reprogramming of development (dauer) or fat metabolism. PRDX-2 is required for longevity at 15 °C, even when IIS is further reduced or the activity of IIS-inhibited transcription factors, DAF-16 or HSF-1 is genetically increased. This suggests that PRDX-2 promotes longevity at 15 °C independently from regulating IIS.

Collectively, these data suggest that the increased intestinal activity of DAF-16 and SKN-1 and arsenite resistance associated with loss of PRDX-2 may be due to impaired insulin secretion (Fig.6). Nevertheless, our data reveal that further reductions in IIS and increases in DAF-16 activity are required for the reprogramming of metabolism and development (Fig.6). Moreover, our data suggest that although increased DAF-16 and SKN-1 activities are able to increase tolerance to an acute stress, their prolongevity activities are unable to fully compensate for the life-shortening effect of loss of PRDX-2 at 15 °C (Fig.6).

## Discussion

Peroxiredoxins are abundant peroxidases that have also been shown to regulate the activity of multiple signalling pathways by stimulating or inhibiting the oxidation of redox-sensitive signalling proteins (Rhee & Woo, 2011). Here, we have identified a new role for the *C. elegans* orthologue of the tumour suppressor Prdx1 in signal transduction through insulin-signalling pathways. We reveal that under nutrient-rich conditions, PRDX-2 promotes IIS by promoting the secretion of the insulin peptide DAF-28.

With increases in type 2 diabetes and cancer associated with deregulated insulin signalling, there is substantial interest in therapeutically manipulating insulin-signalling pathways. It is widely appreciated that ROS have both negative and positive roles in insulin responses (Szypowska & Burgering, 2011). For example, the transient inactivation of the phosphatase PTEN by ROS-induced oxidation has been demonstrated to play an important role in regulating the activity of kinases that participate in the IIS-dependent inhibition of FOXO (Leslie *et al*., 2003). Cytosolic peroxiredoxins have been shown to play an important role in modulating the inactivation of these phosphatases in cultured cells. For example, previous work in mouse cells has suggested that loss of Prdx1 promotes the oxidative inactivation of PTEN and increases Akt activity (Cao *et al*., 2009). Similarly, Prx have also been shown to provide a barrier to the ROS-mediated inactivation of protein tyrosine phosphatases (PTP) during growth factor signalling (Choi *et al*., 2005). These studies in cellular models have revealed several mechanisms by which different peroxiredoxin activities could prevent or promote age-associated diseases, such as diabetes and cancer. For example, by protecting the PTP and PTEN phosphatases from ROS-mediated inactivation, human Prx may provide an important barrier to inappropriate oncogenic signalling (Choi *et al*., 2005; Cao *et al*., 2009). However, Akt kinases also mediate insulin signalling; thus, reduced PTEN or PTP1B activity would be predicted to increase insulin/IGF-1-like signalling. The discovery that a Prx is important for insulin secretion reveals that systemically reducing Prx function may have more complex effects on the insulin-mediated responses of a whole animal than suggested by studies in cell models.

Although it remains to be determined why PRDX-2 is required for normal levels of insulin secretion, it is possible that increased levels of ROS, due to loss of the peroxidase activity of PRDX-2, may inhibit DAF-28::GFP secretion. Consistent with this, the ROS generator, methyl viologen, also inhibits DAF-28::GFP secretion (O. Billing & G. Kao, personal communication). Moreover, changes in the cellular redox environment are also important for the glucose-stimulated release of insulin in mammals (Ivarsson *et al*., 2005; Rebelato *et al*., 2011). Thus, it is possible that the role of PRDX-2 in insulin release could be shared by mammalian Prx. Together, these data suggest that impaired insulin release, and the resulting decrease in IIS, could contribute to the hormetic effects of some stress treatments. Nevertheless, misregulation of IIS can also have a widespread and devastating effect on many cellular functions. For example, high glucose has been shown to reduce *C. elegans* lifespan (Lee *et al*., 2009b; Schlotterer *et al*., 2009). Accordingly, the impaired insulin secretion in *prdx-2-*mutant *C. elegans* also raises the possibility that a reduced ability to promote glucose uptake may contribute to their reduced lifespan.

The effect of loss of PRDX-2 on DAF-28::GFP secretion is comparable to the reported effects of RNAi targeting *asna-1* or mitochondrial components, which have previously been shown to be important for insulin secretion (Figs2 and S1) (Kao *et al*., 2007; Billing *et al*., 2011). However, in contrast to loss of *asna-1* or mitochondrial function, for example *tomm-20*, there is no larval arrest/constitutive dauer phenotype associated with loss of PRDX-2 function. The effect of complete loss of ASNA-1 or mitochondrial function on insulin secretion cannot be quantitated, given the necessity of measuring DAF-28::GFP secretion in adult animals. Hence, it remains possible that the absence of a larval arrest/dauer phenotype associated with loss of PRDX-2 is due to the fact that loss of PRDX-2 does not impair DAF-28::GFP secretion as much as complete loss of ASNA-1 or TOMM-20 function. Alternatively, as discussed below, it could indicate a more specific function for PRDX-2 in the secretion of a particular subset of insulin-like peptides.

The *C. elegans* genome encodes 40 insulin-like proteins that are together involved in the regulation of longevity, stress resistance, metabolism or dauer entry and exit (Li *et al*., 2003; Cornils *et al*., 2011; Ritter *et al*., 2013; Fernandes de Abreu *et al*., 2014; Hung *et al*., 2014). Indeed, it is possible that the increased stress resistance of *prdx-2-*mutant animals reflects impaired secretion of other insulin-like proteins, in addition to DAF-28. DAF-28 inhibits dauer formation and is significantly reduced in dauer stage animals (Li *et al*., 2003). The absence of dauer *prdx-2-*mutant larvae under favourable growth conditions suggests that either secretion of DAF-28 or other insulin-like proteins, such as INS-4 and INS-6 that act together with DAF-28 to inhibit dauer arrest (Cornils *et al*., 2011; Fernandes de Abreu *et al*., 2014; Hung *et al*., 2014), remain sufficient in these animals to suppress the dauer developmental programme under these conditions. It is intriguing that despite impaired secretion of DAF-28 and reduced IIS activity, further changes in IIS are required in order for PRDX-2-deficient animals to undergo dauer development or increased fat synthesis. Recent studies suggest functional redundancies between insulins are common (Ritter *et al*., 2013). Therefore, it is possible that, although DAF-28 secretion is impaired in PRDX-2-deficient animals, levels of other insulins are unaffected and/or sufficient to repress dauer formation and fat metabolism.

Although the increased activity of SKN-1 and DAF-16 in *prdx-2*-mutant animals results in increased stress resistance, our data suggest that PRDX-2 is important for the full life-extending effects of increased SKN-1 and DAF-16 activities. By genetically separating the increased stress resistance associated with increased SKN-1 and DAF-16 activities from their longevity-promoting effects, this provides evidence that the increased stress resistance associated with reduced IIS is not sufficient to increase lifespan. Adaptive, stress-protective, responses may mediate the lifespan extension associated with low levels of ROS (Doonan *et al*., 2008; Yang & Hekimi, 2010; Zarse *et al*., 2012; Martins *et al*., 2014). However, our study supports others, which suggest that the ability to survive an acutely toxic stress does not necessarily correlate with increased lifespan (Doonan *et al*., 2008; Van Raamsdonk & Hekimi, 2012). Understanding the interplay between ROS, signal transduction and ageing in a whole animal is challenging. Our discovery that a peroxiredoxin promotes the release of insulin from neurons to increase the expression of stress defences in other tissues exemplifies the importance of whole animal studies as a vital complement to studies in cellular models.

## Experimental procedures

### *Caenorhabditis elegans* strains

All animals were maintained at 15 °C unless stated otherwise on NGM-Lite agar plates using standard methods. For details of the strains used in this study, please see Data S1 (Supporting information).

### Sodium arsenite sensitivity assays

Approximately 35 well-fed L4 larval stage hermaphrodites were transferred to freshly made NGM-Lite agar plates containing 10 mm sodium arsenite. A ‘pickful’ of OP50 bacteria was transferred to the middle of the plate, and the animals were incubated at 15 °C. The number of dead worms was scored at the indicated time points. Worms that did not respond to a gentle prodding or lacked pharyngeal pumping were scored as dead and removed from the plate. Log-rank survival analysis (Minitab Inc., State College, PA, USA) was used to determine statistically significant differences between groups.

### RNAi

*Escherichia coli* HT115 containing pL4440 (vector control) or pL4440 containing the *prdx-2* or *daf-2* orf were grown overnight at 37 °C to OD_600_ = 1.0 in LB liquid media containing 50 μg mL^−1^ ampicillin (SIGMA-ALDRICH, Poole, UK). 1.0 mm Isopropyl β-D-?1-?thiogalactopyranoside (IPTG) was added to the cultures used to seed RNAi plates [0.3% (w/v) NaCl, 1.7% (w/v) agarose, 0.25% (w/v) bactopeptone, 0.08% (w/v) yeast extract, 1.0 mm CaCl_2_, 1.0 mm MgSO_4_, 2.5 mm phosphate buffer (pH 6.0), 1.0 mm IPTG, 5.0 μg mL^−1^ cholesterol and 60 μg mL^−1^ ampicillin]. Plates were incubated at room temperature for 2 days before use. In Figs3C,D and 4B, RNAi was carried out by microinjection of dsRNA as previously described (Olahova *et al*., 2008).

### Microscopy

Animals were mounted on a 3% agarose pad, anaesthetized with 0.06% levamisole, unless otherwise indicated, and microscopic observations were made using a Zeiss Axioskop 2 (Carl Zeiss Ltd, Welwyn Garden City, UK) or Axiovert microscope (40 or 63× objective lens). GFP was detected by excitation with wavelengths 450–490 nm, and all the images were acquired using axiovision software version 3.1.2.1 (Carl Zeiss Ltd, Welwyn Garden City, UK).

### Analysis of DAF-16 activation

The subcellular localization of DAF-16 was determined in L2/L3 larval stage N2 [DAF-16a::GFP], *prdx-2* (*gk169*) [DAF-16a::GFP] and *age-1* (*hx546*) [DAF-16a::GFP] transgenic animals using a Zeiss Axioskop 2 fluorescent microscope. The DAF-16::GFP localization was scored as cytosolic, intermediate and nuclear depending on major localization of the DAF-16::GFP fusion protein. Images were acquired using axiovision software. Chi-square test (Microsoft Excel) was used to determine statistically significant differences between groups.

### Analysis of levels of SKN-1 in intestinal nuclei

To examine the intestinal expression of SKN-1B/C::GFP or SKN-1op::GFP, SKN-1B/C^*S393A*^::GFP or SKN-1op^*S12A*^::GFP, approximately ten late L4 larval stage animals were transferred to fresh RNAi plates. The F1 progeny produced was scored at L2/L3 larval stage under Zeiss Axioskop 2 fluorescent microscope for the GFP expression in the intestine. Worms were maintained at 15 °C and scored none, low, medium or high, depending on the number of intestinal nuclei in which GFP was visible. ‘None’ refers to no intestinal GFP expression. ‘Low’ indicates that the intestinal GFP expression is present anteriorly, or anteriorly and posteriorly (≤ 7 nuclei). ‘Medium’ indicates GFP expression is present anteriorly and posteriorly in some but not all intestinal nuclei (> 7). ‘High’ refers to animals in which GFP was detectable throughout most of the intestine. Statistically significant differences between groups (*P*-values) were calculated using a chi-square test (Microsoft Excel).

### RNA extraction and quantitative RT–PCR

RNA was extracted from approximately 3000 well-fed age-synchronized wild-type (N2) and *prdx-2* (*gk169*) young adult animals using Trizol (Sigma). The RNA samples were DNaseI treated (Ambion and PrimerDesign Life Technologies, Thermofisher Scientific, Waltham, MA, USA) according to the manufacturer’s instructions and used immediately in the qRT–PCR. Supercript III Platinum SYBR Green One-Step qRT–PCR kit (Invitrogen) or Precision OneStep qRT PCR MasterMix (PrimerDesign) and Corbett Life Science (Qiagen, Crawley, UK) Rotor-Gene 6000 system were used to determine relative levels of *gst-7*, *mtl-1* and *sod-3* gene expression. mRNA levels of *act-1* were used for normalization. A Student’s *t*-test was used to test the statistical significance of differences. Primer sequences are available on request.

### Lifespan analysis

Approximately 15 late L4 larval stage animals were transferred to fresh RNAi plates. The lifespan of F1 progeny produced after approximately 24 h was assessed. Adult hermaphrodites were transferred away from their progeny to a fresh plate every second day until they stopped laying eggs. The lifespan assays were conducted at 15 °C. The viability of each worm was assessed from either L1 larval stage or adulthood. Worms that did not move after repeated prodding with a pick or lacked pharyngeal pumping were scored as dead and removed from the plate. A log-rank survival test (minitab 16) was used to determine whether differences between groups were statistically significant.

### Dauer assay

Approximately 25 young adult animals were allowed to lay eggs for 3 h at 15 °C. The adults were removed, and the eggs were incubated at the indicated temperatures (Table1) for 48 h before dauer formation was scored under a Leica S6 stereomicroscope (Leica, Wetzlar, Germany).

### Analysis of DAF-28::GFP secretion

To assess the secretion of the insulin-like protein DAF-28::GFP from neurons or ssGFP from muscles, the intensity of GFP fluorescence was measured in coelomycetes in 1-day-old adult animals expressing DAF-28::GFP (Kao *et al*., 2007) or ssGFP (Fares & Greenwald, 2001a,b) using a Zeiss Axioskop 2 microscope with excitation at 450–490 nm. Animals were mounted onto microscope slides containing 2% agarose and anesthetized with 0.06% levamisole. All images were taken under the 63× objective lens and captured at the same exposure. The number of coelomocytes in each animal containing visible levels of GFP was scored, and the amount of GFP fluorescence in GFP-positive coelomocytes was quantified using axiovision 3.1.2.1 software. A defined area (± 10%) of a coelomocyte was outlined in each animal, and the mean pixel value and standard error were calculated (Kao *et al*., 2007). A Student’s *t*-test was used to test the statistical significance of differences.

## Supporting Information

**Fig. S1** Loss of PRDX-2 does not reduce the levels of muscle-secreted GFP that accumulate in coelomocytes. **Fig. S2** Loss of PRDX-2 does not affect total or neuronal levels of DAF-28::GFP protein. **Fig. S3** Loss of PRDX-2 increases the intestinal expression of a *sod-3p*::*gfp* reporter in a DAF-2–dependent manner that partially requires DAF-16. **Fig. S4** Loss of PRDX-2 does not reduce IIS sufficiently to increase fat storage. **Fig. S5** PRDX-2 is not required for the extended lifespans associated with reduced CYC-1 levels. **Table S1** Fold induction of *gst-7*, *sod-3* and *mtl-1* mRNA in *prdx-2* mutant compared with wild-type animals. **Table S2** Statistical analysis of Lifespan data for experiments shown in Fig. 5. **Data S1** Supplemental methods including details of strains used in this study.

## References

[b1] Ailion M, Thomas JH (2000). Dauer formation induced by high temperatures in *Caenorhabditis elegans*. Genetics.

[b2] Ailion M, Thomas JH (2003). Isolation and characterization of high-temperature-induced Dauer formation mutants in *Caenorhabditis elegans*. Genetics.

[b3] An JH, Blackwell TK (2003). SKN-1 links *C. elegans* mesendodermal specification to a conserved oxidative stress response. Genes Dev.

[b4] An JH, Vranas K, Lucke M, Inoue H, Hisamoto N, Matsumoto K, Blackwell TK (2005). Regulation of the *Caenorhabditis elegans* oxidative stress defense protein SKN-1 by glycogen synthase kinase-3. Proc. Natl Acad. Sci. USA.

[b5] Billing O, Kao G, Naredi P (2011). Mitochondrial function is required for secretion of DAF-28/insulin in *C. elegans*. PLoS ONE.

[b6] Bluher M, Kahn BB, Kahn CR (2003). Extended longevity in mice lacking the insulin receptor in adipose tissue. Science.

[b7] Cao J, Schulte J, Knight A, Leslie NR, Zagozdzon A, Bronson R, Manevich Y, Beeson C, Neumann CA (2009). Prdx1 inhibits tumorigenesis via regulating PTEN/AKT activity. EMBO J.

[b8] Chen AT, Guo C, Dumas KJ, Ashrafi K, Hu PJ (2013). Effects of *Caenorhabditis elegans* sgk-1 mutations on lifespan, stress resistance, and DAF-16/FoxO regulation. Aging Cell.

[b9] Choi MH, Lee IK, Kim GW, Kim BU, Han YH, Yu DY, Park HS, Kim KY, Lee JS, Choi C, Bae YS, Lee BI, Rhee SG, Kang SW (2005). Regulation of PDGF signalling and vascular remodelling by peroxiredoxin II. Nature.

[b10] Clancy DJ, Gems D, Harshman LG, Oldham S, Stocker H, Hafen E, Leevers SJ, Partridge L (2001). Extension of life-span by loss of CHICO, a *Drosophila* insulin receptor substrate protein. Science.

[b11] Cornils A, Gloeck M, Chen Z, Zhang Y, Alcedo J (2011). Specific insulin-like peptides encode sensory information to regulate distinct developmental processes. Development.

[b12] Doonan R, McElwee JJ, Matthijssens F, Walker GA, Houthoofd K, Back P, Matscheski A, Vanfleteren JR, Gems D (2008). Against the oxidative damage theory of aging: superoxide dismutases protect against oxidative stress but have little or no effect on life span in *Caenorhabditis elegans*. Genes Dev.

[b13] Fares H, Greenwald I (2001a). Genetic analysis of endocytosis in *Caenorhabditis elegans*: coelomocyte uptake defective mutants. Genetics.

[b14] Fares H, Greenwald I (2001b). Regulation of endocytosis by CUP-5, the *Caenorhabditis elegans* mucolipin-1 homolog. Nat. Genet.

[b15] Fernandes de Abreu DA, Caballero A, Fardel P, Stroustrup N, Chen Z, Lee K, Keyes WD, Nash ZM, Lopez-Moyado IF, Vaggi F, Cornils A, Regenass M, Neagu A, Ostojic I, Liu C, Cho Y, Sifoglu D, Shen Y, Fontana W, Lu H, Csikasz-Nagy A, Murphy CT, Antebi A, Blanc E, Apfeld J, Zhang Y, Alcedo J, Ch’ng Q (2014). An insulin-to-insulin regulatory network orchestrates phenotypic specificity in development and physiology. PLoS Genet.

[b16] Gems D, Sutton AJ, Sundermeyer ML, Albert PS, King KV, Edgley ML, Larsen PL, Riddle DL (1998). Two pleiotropic classes of daf-2 mutation affect larval arrest, adult behavior, reproduction and longevity in *Caenorhabditis elegans*. Genetics.

[b17] Henderson ST, Johnson TE (2001). daf-16 integrates developmental and environmental inputs to mediate aging in the nematode *Caenorhabditis elegans*. Curr. Biol.

[b18] Holzenberger M, Dupont J, Ducos B, Leneuve P, Geloen A, Even PC, Cervera P, Le Bouc Y (2003). IGF-1 receptor regulates lifespan and resistance to oxidative stress in mice. Nature.

[b19] Hsu AL, Murphy CT, Kenyon C (2003). Regulation of aging and age-related disease by DAF-16 and heat-shock factor. Science.

[b20] Hu PJ, Xu J, Ruvkun G (2006). Two membrane-associated tyrosine phosphatase homologs potentiate *C. elegans* AKT-1/PKB signaling. PLoS Genet.

[b21] Hung WL, Wang Y, Chitturi J, Zhen M (2014). A *Caenorhabditis elegans* developmental decision requires insulin signaling-mediated neuron-intestine communication. Development.

[b22] Inoue H, Hisamoto N, An JH, Oliveira RP, Nishida E, Blackwell TK, Matsumoto K (2005). The *C. elegans* p38 MAPK pathway regulates nuclear localization of the transcription factor SKN-1 in oxidative stress response. Genes Dev.

[b23] Iraqui I, Kienda G, Soeur J, Faye G, Baldacci G, Kolodner RD, Huang ME (2009). Peroxiredoxin Tsa1 is the key peroxidase suppressing genome instability and protecting against cell death in *Saccharomyces cerevisiae*. PLoS Genet.

[b24] Isermann K, Liebau E, Roeder T, Bruchhaus I (2004). A peroxiredoxin specifically expressed in two types of pharyngeal neurons is required for normal growth and egg production in *Caenorhabditis elegans*. J. Mol. Biol.

[b25] Ivarsson R, Quintens R, Dejonghe S, Tsukamoto K, in ‘t Veld P, Renstrom E, Schuit FC (2005). Redox control of exocytosis: regulatory role of NADPH, thioredoxin, and glutaredoxin. Diabetes.

[b26] Kao G, Nordenson C, Still M, Ronnlund A, Tuck S, Naredi P (2007). ASNA-1 positively regulates insulin secretion in *C. elegans* and mammalian cells. Cell.

[b27] Kenyon C, Chang J, Gensch E, Rudner A, Tabtiang R (1993). A *C. elegans* mutant that lives twice as long as wild type. Nature.

[b28] Kondo M, Yanase S, Ishii T, Hartman PS, Matsumoto K, Ishii N (2005). The p38 signal transduction pathway participates in the oxidative stress-mediated translocation of DAF-16 to *Caenorhabditis elegans* nuclei. Mech. Ageing Dev.

[b29] Lapierre LR, Hansen M (2012). Lessons from *C. elegans*: signaling pathways for longevity. Trends Endocrinol. Metab.

[b30] Lee RY, Hench J, Ruvkun G (2001). Regulation of *C. elegans* DAF-16 and its human ortholog FKHRL1 by the daf-2 insulin-like signaling pathway. Curr. Biol.

[b31] Lee TH, Kim SU, Yu SL, Kim SH, Park DS, Moon HB, Dho SH, Kwon KS, Kwon HJ, Han YH, Jeong S, Kang SW, Shin HS, Lee KK, Rhee SG, Yu DY (2003). Peroxiredoxin II is essential for sustaining life span of erythrocytes in mice. Blood.

[b32] Lee KS, Iijima-Ando K, Iijima K, Lee WJ, Lee JH, Yu K, Lee DS (2009a). JNK/FOXO-mediated neuronal expression of fly homologue of peroxiredoxin II reduces oxidative stress and extends life span. J. Biol. Chem.

[b33] Lee SJ, Murphy CT, Kenyon C (2009b). Glucose shortens the life span of *C. elegans* by downregulating DAF-16/FOXO activity and aquaporin gene expression. Cell Metab.

[b34] Leslie NR, Bennett D, Lindsay YE, Stewart H, Gray A, Downes CP (2003). Redox regulation of PI 3-kinase signalling via inactivation of PTEN. EMBO J.

[b35] Li W, Kennedy SG, Ruvkun G (2003). daf-28 encodes a *C. elegans* insulin superfamily member that is regulated by environmental cues and acts in the DAF-2 signaling pathway. Genes Dev.

[b36] Liao VH, Yu CW (2005). *Caenorhabditis elegans* gcs-1 confers resistance to arsenic-induced oxidative stress. Biometals.

[b37] Lin K, Hsin H, Libina N, Kenyon C (2001). Regulation of the *Caenorhabditis elegans* longevity protein DAF-16 by insulin/IGF-1 and germline signaling. Nat. Genet.

[b38] Lu J, Vallabhaneni H, Yin J, Liu Y (2013). Deletion of the major peroxiredoxin Tsa1 alters telomere length homeostasis. Aging Cell.

[b39] Martins D, Titorenko VI, English AM (2014). Cells with impaired mitochondrial H_2_O_2_ sensing generate less *OH radicals and live longer. Antioxid. Redox Signal.

[b40] Molin M, Yang J, Hanzen S, Toledano MB, Labarre J, Nystrom T (2011). Life span extension and H(2)O(2) resistance elicited by caloric restriction require the peroxiredoxin Tsa1 in *Saccharomyces cerevisiae*. Mol. Cell.

[b41] Morley JF, Morimoto RI (2004). Regulation of longevity in *Caenorhabditis elegans* by heat shock factor and molecular chaperones. Mol. Biol. Cell.

[b42] Murphy CT, McCarroll SA, Bargmann CI, Fraser A, Kamath RS, Ahringer J, Li H, Kenyon C (2003). Genes that act downstream of DAF-16 to influence the lifespan of *Caenorhabditis elegans*. Nature.

[b43] Neumann CA, Krause DS, Carman CV, Das S, Dubey DP, Abraham JL, Bronson RT, Fujiwara Y, Orkin SH, Van Etten RA (2003). Essential role for the peroxiredoxin Prdx1 in erythrocyte antioxidant defence and tumour suppression. Nature.

[b44] Ogg S, Paradis S, Gottlieb S, Patterson GI, Lee L, Tissenbaum HA, Ruvkun G (1997). The Fork head transcription factor DAF-16 transduces insulin-like metabolic and longevity signals in *C. elegans*. Nature.

[b45] Olahova M, Taylor SR, Khazaipoul S, Wang J, Morgan BA, Matsumoto K, Blackwell TK, Veal EA (2008). A redox-sensitive peroxiredoxin that is important for longevity has tissue- and stress-specific roles in stress resistance. Proc. Natl Acad. Sci. USA.

[b46] Oliveira RP, Porter Abate J, Dilks K, Landis J, Ashraf J, Murphy CT, Blackwell TK (2009). Condition-adapted stress and longevity gene regulation by *Caenorhabditis elegans* SKN-1/Nrf. Aging Cell.

[b47] Perez CL, Van Gilst MR (2008). A 13C isotope labeling strategy reveals the influence of insulin signaling on lipogenesis in *C. elegans*. Cell Metab.

[b48] Rebelato E, Abdulkader F, Curi R, Carpinelli AR (2011). Control of the intracellular redox state by glucose participates in the insulin secretion mechanism. PLoS ONE.

[b49] Rhee SG, Woo HA (2011). Multiple functions of peroxiredoxins: peroxidases, sensors and regulators of the intracellular messenger HO, and protein chaperones. Antioxid. Redox Signal.

[b50] Ritter AD, Shen Y, Fuxman Bass J, Jeyaraj S, Deplancke B, Mukhopadhyay A, Xu J, Driscoll M, Tissenbaum HA, Walhout AJ (2013). Complex expression dynamics and robustness in *C. elegans* insulin networks. Genome Res.

[b51] Schlotterer A, Kukudov G, Bozorgmehr F, Hutter H, Du X, Oikonomou D, Ibrahim Y, Pfisterer F, Rabbani N, Thornalley P, Sayed A, Fleming T, Humpert P, Schwenger V, Zeier M, Hamann A, Stern D, Brownlee M, Bierhaus A, Nawroth P, Morcos M (2009). *C. elegans* as model for the study of high glucose- mediated life span reduction. Diabetes.

[b52] Seo K, Choi E, Lee D, Jeong DE, Jang SK, Lee SJ (2013). Heat shock factor 1 mediates the longevity conferred by inhibition of TOR and insulin/IGF-1 signaling pathways in *C. elegans*. Aging Cell.

[b53] Szypowska AA, Burgering BM (2011). The peroxide dilemma: opposing and mediating insulin action. Antioxid. Redox Signal.

[b54] Tullet JM, Hertweck M, An JH, Baker J, Hwang JY, Liu S, Oliveira RP, Baumeister R, Blackwell TK (2008). Direct inhibition of the longevity-promoting factor SKN-1 by insulin-like signaling in *C. elegans*. Cell.

[b55] Van Raamsdonk JM, Hekimi S (2012). Superoxide dismutase is dispensable for normal animal lifespan. Proc. Natl Acad. Sci. USA.

[b56] Yang W, Hekimi S (2010). A mitochondrial superoxide signal triggers increased longevity in *Caenorhabditis elegans*. PLoS Biol.

[b57] Zarse K, Schmeisser S, Groth M, Priebe S, Beuster G, Kuhlow D, Guthke R, Platzer M, Kahn CR, Ristow M (2012). Impaired insulin/IGF1 signaling extends life span by promoting mitochondrial L-proline catabolism to induce a transient ROS signal. Cell Metab.

