# Supplementary material for: A peroxiredoxin, PRDX-2, is required for insulin secretion and insulin/IIS-dependent regulation of stress resistance and longevity
Source: Aging Cell. 2015 Mar 23;14(4):558–68. doi: 10.1111/acel.12321 (PMC4531070; doi:10.1111/acel.12321)
Supplement: Supplementary file 1 [file acel0014-0558-sd1.pdf]

Fig. S1 Loss of PRDX-2 does not reduce the levels of muscle-secreted GFP that accumulate in coelomocytes. 100% of control and *prdx-2* RNAi-treated animals expressing a soluble, secreted GFP (ssGFP) from a muscle-specific promoter (*myo-3p*::ssGFP) contained 6 GFP-positive coelomocytes (data not shown) and **[A]** the intensity of GFP fluorescence in these coelomocytes was not reduced by *prdx-2* RNAi whereas **[B]** *prdx-2* RNAi substantially reduced the number of coelomocytes containing visible levels of GFP in wild type (N2) expressing DAF-28::GFP (chi^2test: control vs *prdx-2* RNAi-treated $p=5.82x10^{-7}$).

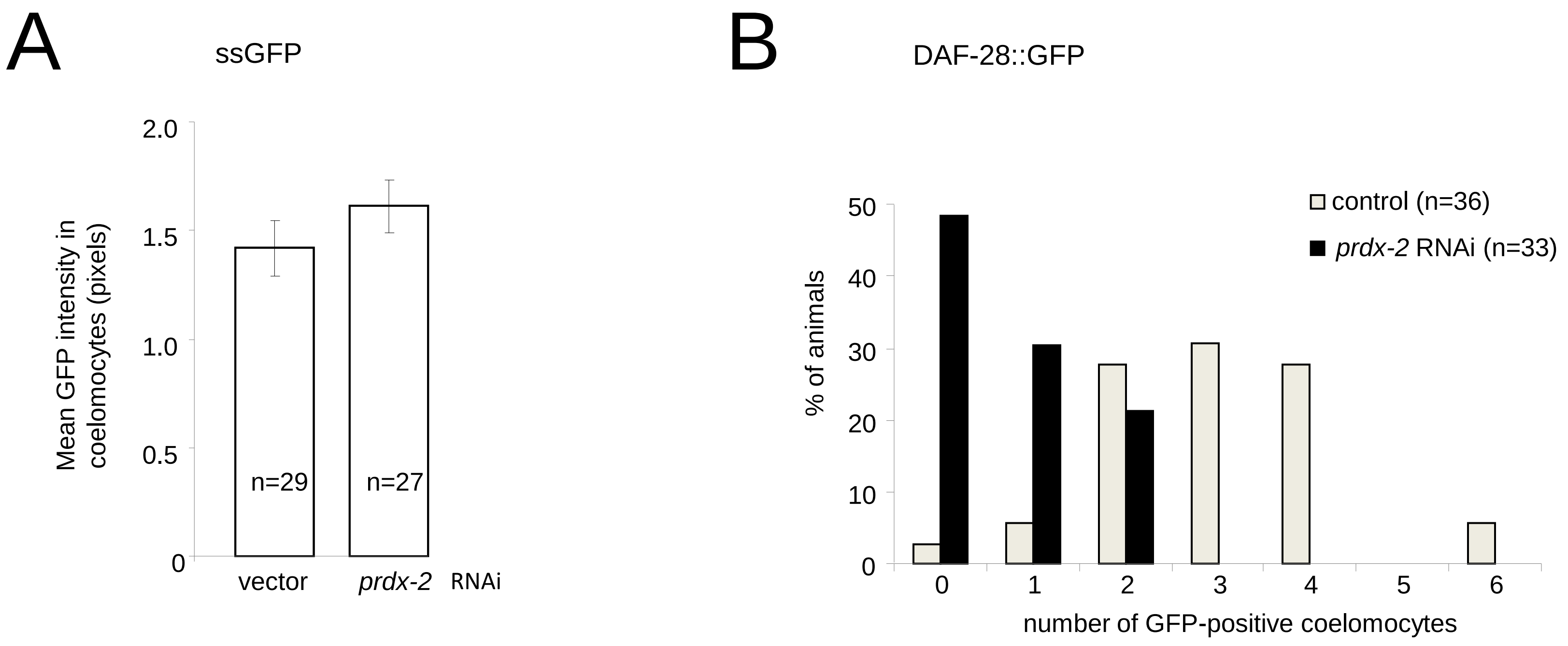

Fig. S2 Loss of PRDX-2 does not affect total or neuronal levels of DAF-28::GFP protein. [A] Representative fluorescent images taken (under the 63x lens) with identical exposures of the heads of 6 animals, and **[B]** quantification of the GFP fluorescence intensity, reveal similar levels of DAF-28::GFP present in the ASJ or ASI neurones of control and *prdx-2* RNAi-treated 1 day old adult N2 DAF-28::GFP animals. DAF-28::GFP fluorescence was quantified in the brightest neurone (indicated by arrow head) by measuring the mean pixel density in fluorescent images of vector control and *prdx-2* RNAi treated 1 day old adults with identical exposures. Error bars represent the standard deviation from the mean. (n) refers to number of animals in each group. The 8% increase in neuronal DAF-28::GFP in *prdx-2* RNAi-treated animals is consistent with a secretion defect but not statistically significant (T test; p=0.138) **[C]** Immunoblotting with anti-GFP antibodies reveals that wild type (N2) and *prdx-2* (*gk169*) mutant animals expressing a DAF-28::GFP transgene contain similar amounts of DAF-28::GFP protein. Non-specific bands that are also present in wild type (N2) animals that do not contain the transgene are indicated (ns) and confirm even protein loading. Image J quantification of DAF-28::GFP and 'ns' bands was used to normalise DAF-28::GFP levels and the relative levels of DAF-28::GFP in wild-type and *prdx-2* mutant animals are indicated. Experiment was repeated with similar results.

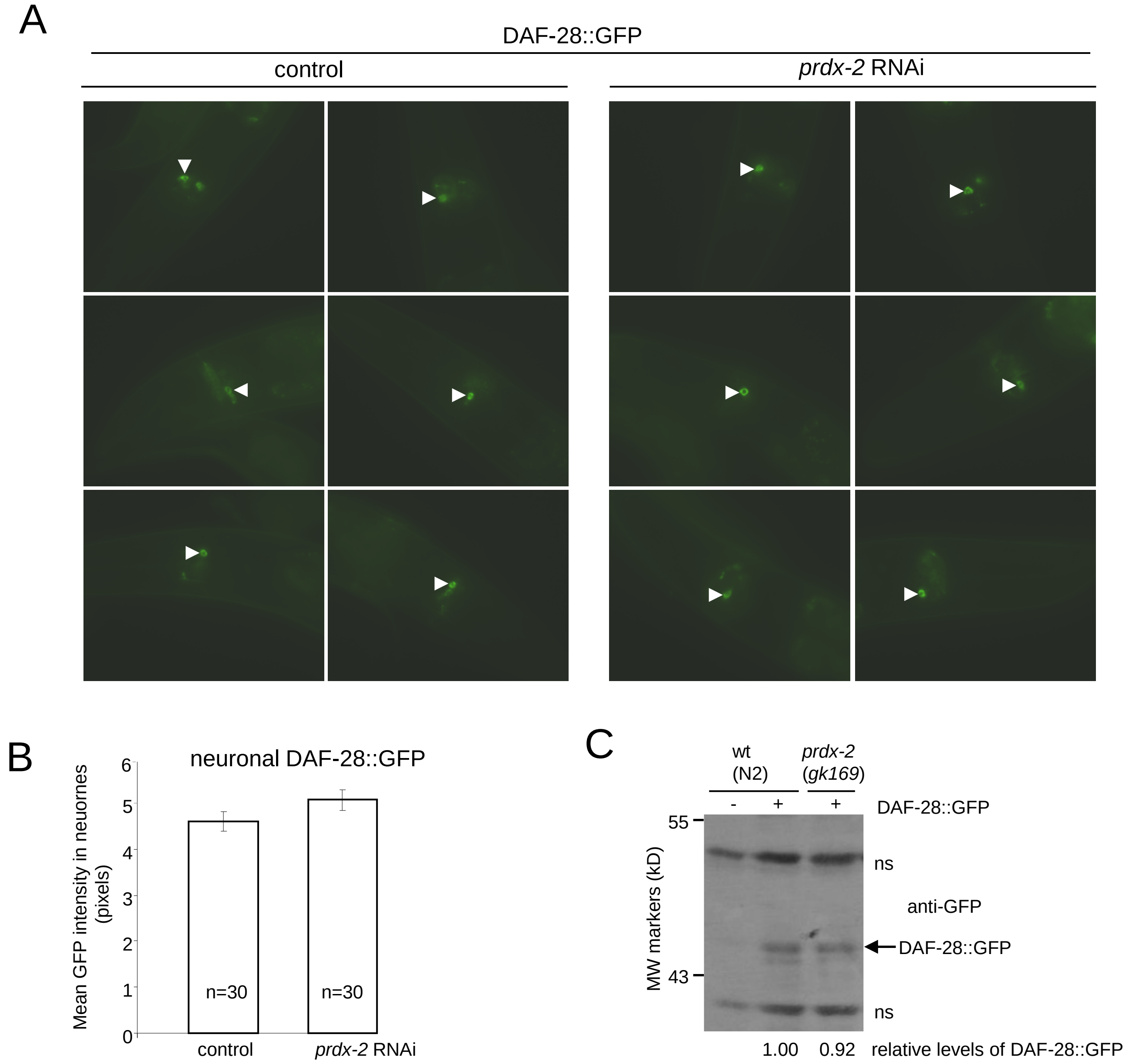

Fig. S3 Loss of PRDX-2 increases the intestinal expression of a *sod-3p::gfp* reporter in a DAF-2–dependent manner that partially requires DAF-16. **[A]** Representative images of control , *prdx-2* and *daf-2* RNAi-treated N2 *sod-3p::gfp* animals. **[B]** Scoring of *sod-3p::gfp* revealed that **[C]** *prdx-2* RNAi significantly increased the intestinal expression of *sod-3p::gfp* (Chi^2 test p= 0.0075) but to a lesser extent than *daf-2* RNAi (Chi^2 test p= 1.1 x10^{-14}) in N2 *sod-3p::gfp* **[D]** *prdx-2* and *daf-2* RNAi cause much smaller increases in *sod-3p::gfp* expression in *daf-16* (*mu86*) *sod-3p::gfp* animals (although still significant; Chi^2 test p<0.001), and *prdx-2* RNAi does not cause any further increase the intestinal expression of *sod-3p::gfp* in *daf-2* (*e1370*) animals.

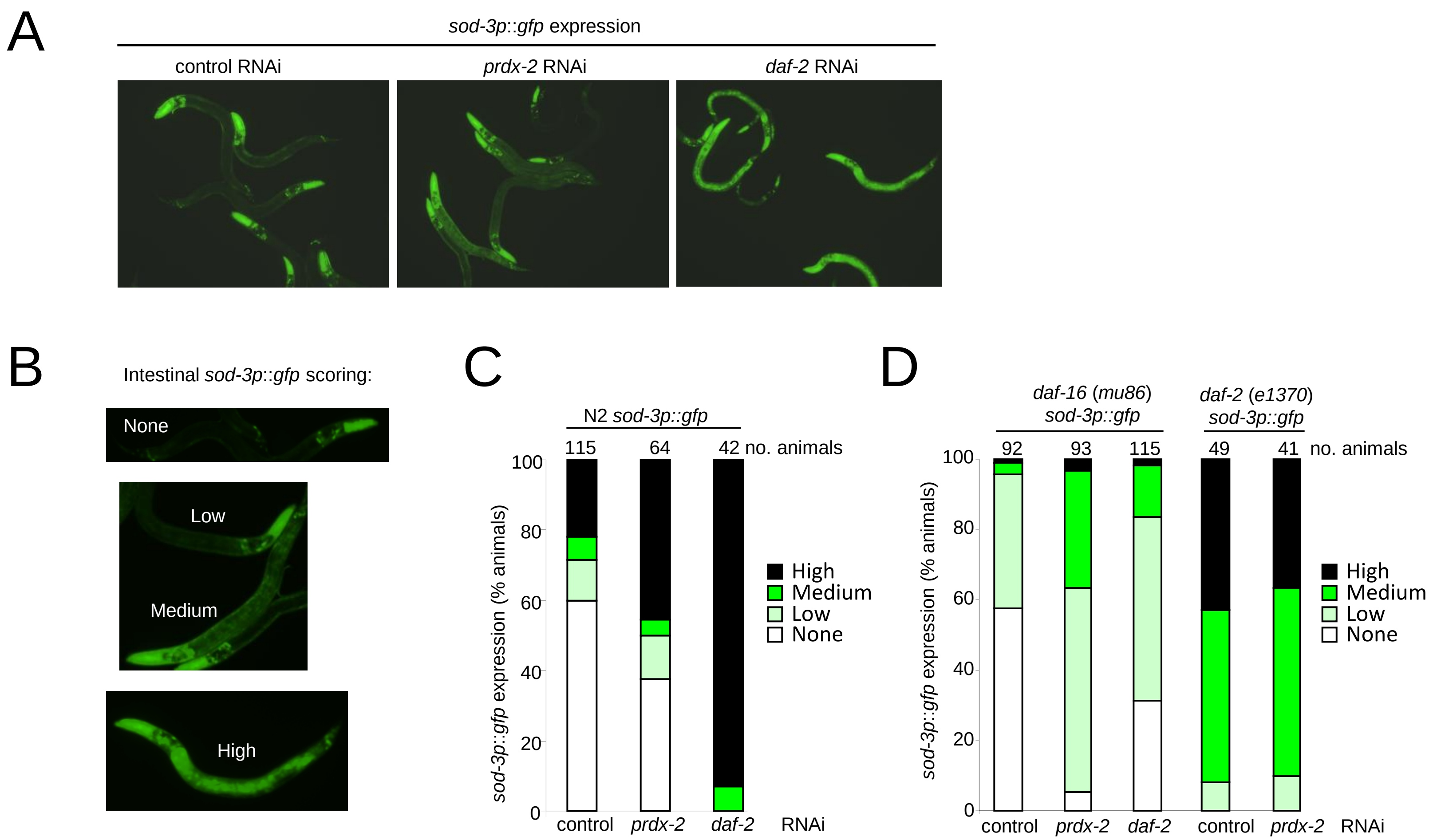

Fig.S4 Loss of PRDX-2 does not reduce IIS sufficiently to increase fat storage. Body fat content was visualized in wild type (N2), *prdx-2* (*gk169*), *daf-2* (*e1370*) and *prdx-2* (*gk169*) *daf-2* (*e1370*) double mutant animals using Sudan black staining (Ogg & Ruvkun 1998). This revealed that *prdx-2* mutant and wild type animals contain similar levels of fat and that loss of PRDX-2 does not affect the increased fat storage associated with reduced DAF-2 activity in *daf-2* (*e1370*) mutant animals. The experiment was repeated 3 times yielding similar results. Representative animals are shown.

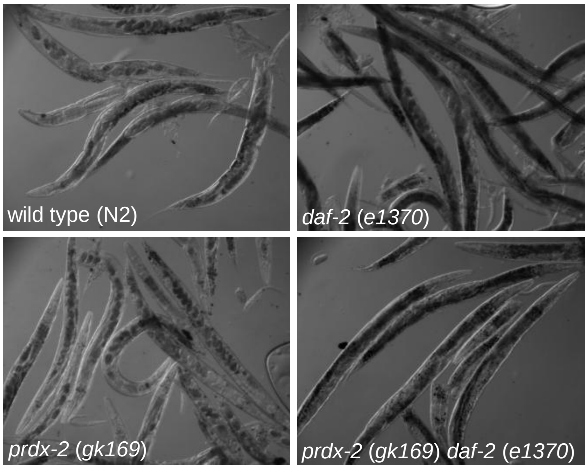

Fig. S5 PRDX-2 is not required for the extended lifespans associated with reduced CYC-1 levels. The lifespan of wild type and *prdx-2* (*gk169*) mutant animals on control and *cyc-1* RNAi. *Cyc-1* RNAi extended the lifespan of both wild type (N2) and *prdx-2* mutant animals. The similar lifespan of wild type (N2) and *prdx-2* (*gk169*) mutants on *cyc-1* RNAi suggests that PRDX-2 is not required for the increased lifespan associated with reduced mitochondrial activity (Dillin et al. 2002). This experiment was repeated with similar results and a representative experiment is shown.

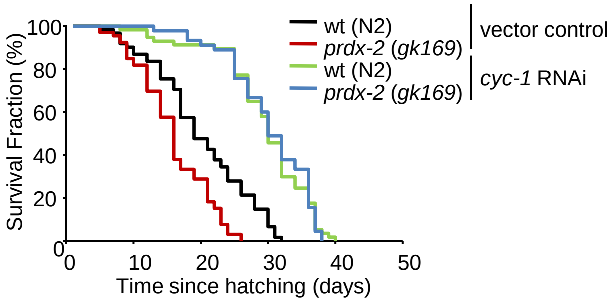

Table S1 Fold Induction of *gst-7*, *sod-3* and *mtl-1* mRNA in *prdx-2* mutant compared with wild-type animals. Each experiment represents an independent biological repeat in which the fold induction of *gst-7*, *sod-3* and *mtl-1* mRNA was determined from the mean of 3-5 technical replicate mRNA measurements (relative to mean *act-1* mRNA levels) in RNA samples independently isolated from wild-type and *prdx-2* mutant animals. Although there was some variation in the fold induction between individual experiments, in every experiment there was an increase in the expression of *gst-7*, *sod-3* and *mtl-1* compared with wild-type animals. A Student's T test was used to determine the statistical significance of differences between the levels of *gst-7*, *sod-3* or *mtl-1* mRNA in wild-type and *prdx-2* mutant *C. elegans*.

***gst-7* mRNA**	Expt 1	Expt 2	Expt3	Expt 4	Expt 5	Expt 6		mean	SEM	T test
wild-type (N2)	1	1	1	1	1	1				
prdx-2 (*gk169*)	11.74	5.97	2.91	3.20	6.84	2.57		5.54	1.43	0.025
***sod-3* mRNA**	Expt 1	Expt 2	Expt 3	Expt 4	Expt 5	Expt 6	Expt 7			
wild-type (N2)	1	1	1	1	1	1	1			
prdx-2 (*gk169*)	1.65	1.65	8.45	1.25	1.45	1.62	2.94	2.72	0.91	0.13
***mtl-1* mRNA**	Expt 1	Expt 2	Expt 3	Expt 4	Expt 5	Expt 6				
wild-type (N2)	1	1	1	1	1	1				
prdx-2 (*gk169*)	3.7	4.9	8.01	11.25	2.13	12.17		7.03	1.68	0.016

Table S2 Statistical analysis of Lifespan data for experiments shown in Fig.5

Figure 5A:

	**RNAi**	***n***	**Median [days]**	**Mean ± SEM [days]**	***p*[¥]**	***p*[¥¥]**	***p*[¥¥¥]**
**wild type (N2)**	*control*	128	26	24.74 ± 0.553	-	<0.0001	
daf-2 **(*e1370*)**	*control*	140	33	32.31± 0.651	<0.0001	<0.0001	
**wild type (N2)**	*prdx-2*	128	18	18.52 ± 0.522	<0.0001	-	
daf-2 **(*e1370*)**	*prdx-2*	114	18	19.03 ± 0.540	<0.0001	0.901	<0.0001

p[¥] - p value [cf with N2 control]
p[¥¥] - p value [cf with N2 + *prdx-2* RNAi]
p[¥¥¥] - p value [cf with *daf-2 (e1370*) control]

Figure 5B:

	**RNAi**	***n***	**Median [days]**	**Mean ± SEM [days]**	***p*[¥]**	***p*[¥¥]**	***p*[¥¥¥]**
**wild type (N2)**	*control*	73	18	17.14 ± 0.801	-	<0.0001	
sgk-1 **(*ft15*)**	*control*	103	24	21.83 ± 1.010	<0.0001	<0.0001	
**wild type (N2)**	*prdx-2*	60	13	12.13 ± 0.523	<0.0001	-	
sgk-1 **(*ft15*)**	*prdx-2*	93	14	13.67 ± 0.371	<0.0001	0.019	<0.0001

p[¥] - p value [cf with N2 control]
p[¥¥] - p value [cf with N2 + *prdx-2* RNAi]
p[¥¥¥] - p value [cf with *sgk-1 (ft15)* control]

Figure 5C:

	**RNAi**	***n***	**Median [days]**	**Mean ± SEM [days]**	***p*[¥]**	***p*[¥¥]**	***p*[¥¥¥]**
**wild type (N2)**	*control*	64	18	16.84 ± 0.655	-	<0.0001	
hsf-1oe	*control*	54	25	22.72 ± 1.070	<0.0001	<0.0001	
**wild type (N2)**	*prdx-2*	81	13	12.26 ± 0.484	<0.0001	-	
hsf-1oe	*prdx-2*	78	13	11.86 ± 0515	<0.0001	0.635	<0.0001

p[¥] - p value [cf with N2 control]
p[¥¥] - p value [cf with N2 + *prdx-2* RNAi]
p[¥¥¥] - p value [cf with *hsf-1 oe* control]

Supplemental methods

***C. elegans* strains**

N2 Bristol as wild type, VE1: *prdx-2* (*gk169*) II, CB1370: *daf-2* (*e1370*) III, VE14: *prdx-2* (*gk169*) II; *daf-2* (*e1370*) III, CF1038: *daf-16* (*mu86*) I, EU1: *skn-1* (*zu67*) IV, KQ1654: *sgk-1* (*ft15*), BQ1: *akt-1* (*mg306*) V, CF1824: *muEx265* [*HSF-1p::HSF-1cDNA +myo-3::GFP*], CF1407: *daf-16* (*mu86*) I; *muIs71* [pKL99(*daf-16Ap::GFP::daf-16A*(bKO)) + pRF4(*rol-6*)], XA2974: *age-1(hx546) daf-16* (*mu86*) I; *muIs71* [pKL99(*daf-16Ap::GFP::daf-16A*(bKO)) + pRF4(*rol-6*)], VE15: *prdx-2* (*gk169*) II *daf-16* (*mu86*) I; *muIs71* [pKL99(*daf-16Ap::GFP::daf-16A*(bKO)) + pRF4(*rol-6*)], CF1553: *muIs84* [(pAD76) *sod-3p::GFP* + *rol-6*], CF1874: *daf-16*(*mu86*) I; *muIs84* [(pAD76) *sod-3p::GFP* + *rol-6*], CF1580: *daf-2*(*e1370*) III; *muIs84* [(pAD76) *sod-3p::GFP* + *rol-6*], LD1257: N2 *ldEx010* [SKN-1op::GFP], LD1250: N2 *ldEx007* [SKN-1B/C::GFP], LD1256: N2 *ldEx015* [SKN -1op^{S12A}::GFP], LD1252: N2 *ldEx020* [SKN-1B/C^{S393A}::GFP], GS1912: *arIs37* [*myo-3p::ssGFP +dpy-20*(+)] I; *dpy-20*(*e1282*) IV, VB1605: *unc-4* (*e120*) II; *svIs69* [(*daf-28p::daf-28*::*GFP*) + *unc-4* (+)]. The continued presence of the *unc-4* (*e120*) allele in the backcrossed VB1605 was unconfirmed. VE16: *prdx-2* (*gk169*) *unc-4* (*e120*) II; svIs69 [(*Pdaf-28::daf-28::GFP*) + *unc-4* (+)]. VE14 was obtained from a cross between VE1 and CB1370. VE15 was obtained from a cross between VE1 and CF1407. VE16 was obtained from a cross between VE1 and VB1605. Strains were maintained using standard methods (Brenner 1974).

Analysis of DAF-28::GFP levels by immunoblotting

Approximately 5000 synchronised 1 day old adult worms (N2, VB1605 or VE16) maintained on HT115 containing pL4440 were harvested and proteins extracted under native conditions, basically as described previously (Olahova *et al.* 2008). 40µg of reduced,

denatured protein was loaded into each lane and analysed by immunoblotting using 1in 2000 diluted rabbit anti-GFP primary antibodies (Molecular Probes), HRP-conjugated anti-rabbit antibodies, chemiluminescent (ECL) detection and X ray film (Fuji).

Analysis of *sod-3p::gfp* expression

To determine the expression of *sod-3p::gfp* 10 late L4 larval stage wild-type (N2), *daf-2* (*e1370*) or *daf-16* (*mu86*) animals containing the *sod-3p::gfp* reporter gene (CF1553, CF1580 or CF1874 respectively) were transferred to RNAi plates seeded with control pL4440, pL4440 + *prdx-2* or pL4440 + *daf-2* containing *E. coli HT115*. Animals were maintained at 15°C and *sod-3p::gfp* expression analysed in young adult (N2) or L2/3 larval (*daf-2* or *daf-16*) F1 progeny. *Sod-3p::gfp* expression in the intestine was scored as none, low (20% of cells contain GFP), medium (more than 20% of cells contain GFP) or high (all cells contain GFP). Images were acquired using Axiovision 3.1and a chi^2 test (Microsoft Excel) used to determine whether differences were statistically significant.

Sudan Black fat staining

Well-fed animals were washed in M9 buffer for 30 minutes, fixed in 1% (w/v) paraformaldehyde, followed by three freeze/thaw cycles. After fixation worms were subjected to 3 sequential washes in 25%, 50% and 70% ethanol. The worms were stained overnight in a saturated solution of Sudan black in 70% ethanol, re-hydrated through series of ethanol washes (70%-50%-25%) and the fat droplets were visualised by light microscopy using a Zeiss Axioskop 2 (10x objective lens).

Supplementary references

Brenner, S. (1974). The genetics of Caenorhabditis elegans. Genetics *77*, 71-94.

Dillin A, Hsu AL, Arantes-Oliveira N, Lehrer-Graiwer J, Hsin H, Fraser AG, Kamath RS, Ahringer J, Kenyon C (2002). Rates of behavior and aging specified by mitochondrial function during development. Science. *298*, 2398-2401.

Ogg, S., and Ruvkun, G. (1998). The C. elegans PTEN homolog, DAF-18, acts in the insulin receptor-like metabolic signaling pathway. Mol Cell *2*, 887-893.
